# Down-Regulation of Yeast Helicase Ded1 by Glucose Starvation or Heat-Shock Differentially Impairs Translation of Ded1-Dependent mRNAs

**DOI:** 10.3390/microorganisms9122413

**Published:** 2021-11-23

**Authors:** Neelam Dabas Sen, Hongen Zhang, Alan G. Hinnebusch

**Affiliations:** 1 Division of Molecular and Cellular Biology, Eunice K. Shriver National Institute of Child Health and Human Development, National Institutes of Health, Bethesda, MD 20892, USA; hzhang@mail.nih.gov; 2 School of Life Sciences, Jawaharlal Nehru University, New Delhi 110067, India

**Keywords:** Ded1/DDX3X, RNA helicase, translation, glucose starvation, heat-shock, eIF4B, eIF4A

## Abstract

Ded1 is an essential DEAD-box helicase in yeast that broadly stimulates translation initiation and is critical for mRNAs with structured 5′UTRs. Recent evidence suggests that the condensation of Ded1 in mRNA granules down-regulates Ded1 function during heat-shock and glucose starvation. We examined this hypothesis by determining the overlap between mRNAs whose relative translational efficiencies (TEs), as determined by ribosomal profiling, were diminished in either stressed WT cells or in *ded1* mutants examined in non-stress conditions. Only subsets of the Ded1-hyperdependent mRNAs identified in *ded1* mutant cells exhibited strong TE reductions in glucose-starved or heat-shocked WT cells, and those down-regulated by glucose starvation also exhibited hyper-dependence on initiation factor eIF4B, and to a lesser extent eIF4A, for efficient translation in non-stressed cells. These findings are consistent with recent proposals that the dissociation of Ded1 from mRNA 5′UTRs and the condensation of Ded1 contribute to reduced Ded1 function during stress, and they further suggest that the down-regulation of eIF4B and eIF4A functions also contributes to the translational impairment of a select group of Ded1 mRNA targets with heightened dependence on all three factors during glucose starvation.

## 1. Introduction

Most eukaryotic mRNAs are translated by the scanning mechanism, which begins with the assembly of a 43S preinitiation complex containing the small (40S) ribosomal subunit loaded with the Met-tRNA_i_^Met^ initiator and several initiation factors (eIFs). The 43S PIC binds to the 5′-end of mRNA, with eIF4F bound to the m^7^G cap (comprised of cap-binding protein eIF4E, scaffolding subunit eIF4G, and DEAD-box RNA helicase eIF4A), and scans the 5′-untranslated region (UTR) to identify the start codon. The 48S PIC joins with the large (60S) subunit to form an 80S initiation complex ready to begin protein synthesis (reviewed in [1,2]). Both 43S PIC attachment to mRNA and subsequent scanning are inhibited by secondary structures in the mRNA 5′UTR [3] that are thought to be resolved by DEAD/H-box RNA helicases. The association of eIF4A with eIF4F should facilitate the unwinding of cap-proximal structures; however, other helicases, including Dhx29 in mammals and Ded1/Ddx3 in budding yeast, are additionally required to resolve cap-distal structures that impede scanning (reviewed in [4,5]).

eIF4A is required for 48S PIC assembly in vitro for all mRNAs tested regardless of their structural complexity [6,7]. Evidence suggests that mammalian eIF4A remodels the 40S subunit to enhance PIC attachment [8] and might facilitate the threading of the 5′-end of mRNA into the 40S entry channel [9]. The ribosome footprint profiling of a yeast eIF4A temperature-sensitive mutant (*tif1-ts)* revealed that, despite a strong reduction in bulk polysome assembly, the inactivation of eIF4A markedly reduced the relative translational efficiencies (TEs) of less than 40 mRNAs [10], suggesting that the majority of mRNAs have similar requirements for eIF4A in yeast cells. In contrast, a comparable inactivation of Ded1 in a cold-sensitive Ded1 mutant (*ded1-cs*) was found to reduce the relative TEs of >1100 mRNAs, and these Ded1-hyperdependent mRNAs displayed a marked tendency for long, structure-prone 5′UTRs, suggesting that Ded1 plays a larger role than eIF4A in promoting the translation of mRNAs harboring stable 5’UTR structures [5,10]. This mRNA-specific role was even more evident when Ded1 was inactivated in a mutant lacking the closely related paralog Dbp1. The profiling of 40S subunits showed that both the attachment of 43S PICs and subsequent scanning to the AUG start codon were preferentially impaired on Ded1-hyperdependent mRNAs in *ded1-cs* and *ded1-ts dbp1Δ* mutants [5]. The heightened requirement for Ded1 to stimulate the assembly of 48S PICs on Ded1-hyperdependent mRNAs with structured 5′UTRs was reconstituted in a purified yeast translation initiation system [11]. Interestingly, the Ded1 stimulation of 48S PIC assembly in this system was enhanced by eIF4F and domains in eIF4G and Ded1 that mediate Ded1-eIF4F association [11], including Ded1 N-terminal amino acids necessary for its interactions with eIF4A and eIF4E [12]. These last findings supported previous biochemical evidence that Ded1 unwinding activity on a model substrate is stimulated by eIF4A and eIF4G [13].

There is evidence that Ded1 can also repress the translation of mRNAs by segregating them into insoluble RNA–protein complexes during stress, including processing bodies (PBs) and stress granules (SGs). Ded1 is a PB component formed in response to glucose starvation (−Glu) or oxidative stress, and the genetic depletion of Ded1 reduces PB formation under such stress conditions while reducing bulk translation in non-stressed cells, whereas the overexpression of Ded1 inhibits cell growth and induces PB formation in non-stressed cells [14]. Ded1 ATPase activity appears to oppose SG formation, while regions in the Ded1 N-terminal domain (NTD) and C-terminal domain (CTD) contribute to SG formation by overexpressed Ded1. These domains also stimulate translation at native levels of Ded1 expression [15] and mediate its interaction with eIF4A [12] or eIF4G [15]. It was proposed that Ded1 modulates translation by initially forming a complex with eIF4F and mRNA that is stalled in translation initiation upstream of 43S joining and subsequently utilizes ATP hydrolysis to allow for the stalled complex to progress into the initiation pathway. The overexpression of WT Ded1 or the inactivation of its ATPase activity would impede the transition from stalled to translationally productive mRNPs and lead to Ded1 sequestration with the bound mRNAs in SGs [15].

More recently, Weis et al. reported that purified Ded1 undergoes phase separation into liquid droplets in a manner reversed by the activation of its ATPase activity by a segment of eIF4G [16]. Furthermore, the expression of the catalytic inactive ded1-DQAD variant leads to constitutive SG granule formation and prolongs the lifetime of SGs formed by glucose starvation on treatment with cycloheximide. These and other findings [17] suggest that Ded1 is an active participant in SG formation when its ATPase activity is impaired, and it functionally interacts with another DEAD-box helicase Dhh1 required for PB formation in the progression of translationally inactive mRNAs from PBs to SG during glucose starvation [16].

Heat-shock, in addition to glucose starvation, evokes the rapid sequestering of Ded1 into SGs [18] that increases with temperature and is correlated with reduced growth [19]. Condensation appears to be antagonized by a polar segment in the Ded1 NTD predicted to be an intrinsically disordered region (IDR; residues 35–53), which appears to minimize sequestration at low temperatures and promote cell survival at high temperatures, presumably by preventing the excessive sequestration of Ded1 and associated mRNAs in SGs during stress. Purified Ded1 was found to exhibit heat-induced phase separation into gel-like condensates in a manner enhanced by mRNA, which requires the Ded1 CTD and is enhanced at lower temperatures by substituting the N-terminal IDR. Interestingly, heat-shock selectively reduced the TEs of mRNAs that tend to have 5′UTRs with greater than average propensity for structure formation [19], as was previously shown for the mutational inactivation of Ded1 [5,10]. The *DED1-IDRm* mutation disfavors the translation of mRNAs with these properties (at least at the high temperature of 42 °C), and it also increases the enrichment of such mRNAs in the insoluble fraction formed in cells. Thus, it was proposed that the sequestration of Ded1 and associated mRNAs in SGs during heat-shock preferentially reduces the translation of mRNAs with structurally complex 5′UTRs that heavily depend on Ded1 unwinding activity for translation [19]. It was not explicitly shown, however, that the mRNAs whose TEs are reduced during the heat-shock of WT cells, or those preferentially impaired by the *DED1-IDRm* mutation during a strong heat-shock of 42 °C, are enriched for mRNAs shown to be Ded1-hyperdependent via the ribosome profiling of *ded1* mutants [5,10].

Recent results have indicated that the withdrawal of glucose elicits a rapid loss of binding by Ded1 and initiation factors eIF4A and eIF4B from the translation initiation regions of many mRNAs, without a commensurate dissociation of Ded1 from within mRNA bodies [20]; these results are consistent with previous findings for eIF4A [21]. A more gradual decline in mRNA association by the same three proteins was observed in heat-shock, which was more pronounced for Ded1 and accompanied by the dissociation of eIF4G, although mRNA degradation might contribute to the loss of protein–mRNA interactions during heat stress [20]. These findings seem consistent with the rapid down-regulation of Ded1 function in unwinding 5′UTR structures that reduces initiation rates, which might be accompanied by the sustained association of Ded1 with mRNA-coding sequences within SGs formed on glucose starvation or heat-shock [19].

Interestingly, the deletion of the Ded1 CTD was reported to enhance cell growth when the TORC1 kinase complex is inhibited by rapamycin, and this growth phenotype, as well as increased bulk translation, was conferred by the elimination of the last 14 residues of Ded1. As these CTD mutations also impaired Ded1 interaction with eIF4G, it was proposed that Ded1 association with eIF4G is instrumental in down-regulating translation upon inhibition of TORC1 [22]. Other evidence has suggested that the enhanced degradation of eIF4G, shown to be triggered by rapamycin [23], is mediated by eIF4G’s interaction with Ded1, is dependent on Ded1 helicase activity (presumably to dissociate eIF4G from target mRNAs), and provides an important mechanism for reducing the translation initiation of TORC1 inhibition by rapamycin [22]. However, it was not determined whether mRNAs translationally impaired by the inhibition of TORC1 exhibit a heightened dependence on eIF4G for their translation in non-stressed cells replete with active TORC1.

We wished to test the model in which the sequestration of Ded1 in RNA granules mediates the down-regulation of Ded1 function and the sequestration of Ded1-hyperdependent mRNAs in insoluble aggregates and, as such, represents an important driver of the reduced translation of mRNAs with heightened dependence on Ded1 during cellular stress. To this end, we interrogated ribosome-profiling data on WT cells subjected to glucose starvation or heat-shock and asked whether the TE reductions in these conditions are substantially enriched for those conferred by inactivating *ded1* mutations in non-stressed cells or by the *DED1-IDRm* allele during heat stress. We also examined whether TE changes conferred by the Rap treatment of WT cells reflects the down-regulation of Ded1 or eIF4G function in response to the reduced activity of TORC1. Our analysis supports the idea that Ded1 function is decreased in glucose-starved and heat-shocked cells, but only a subset of Ded1-hyperdependent mRNAs were found to show marked TE reductions, suggesting contributions from additional factors, including eIF4B and eIF4A, in down-regulating their translation.

## 2. Methods

### Analysis of Ribosome Footprint Profiling and RNA-Seq Datasets

Ribosome-profiling datasets from yeast cells were obtained from eight independent studies, and the GEO database series and sample accession numbers of all analyzed datasets are listed in Table S1. For the GSE51532, GSE67387, GSE131176 and GSE141029, datasets, the count data were directly retrieved from the GEO database. For GSE56622, GSE87614, GSE111255, GSE66411 and GSE81966, fastq records were retrieved from GEO and processed with our custom pipeline that includes adaptor trimming, the removal of rRNA reads, and alignment to the genome [24]. We obtained RFP and mRNA read counts using the R package RiboProR (https://github.com/hzhanghenry/RiboProR (accessed on 21 November 2021)). The statistical analysis of differences in ribosome footprint or RNA-seq read counts, or TE values, between WT and mutant samples from biological replicates was primarily conducted using DESeq2 [25], with one case also conducted with Xtail [26]. Genes with less than 128 of total mRNA reads in the 4 samples combined (two replicates of both WT and mutant strains, or treated versus untreated WT cells, under comparison) were excluded from the calculation of TE values.

**Figure 1:**
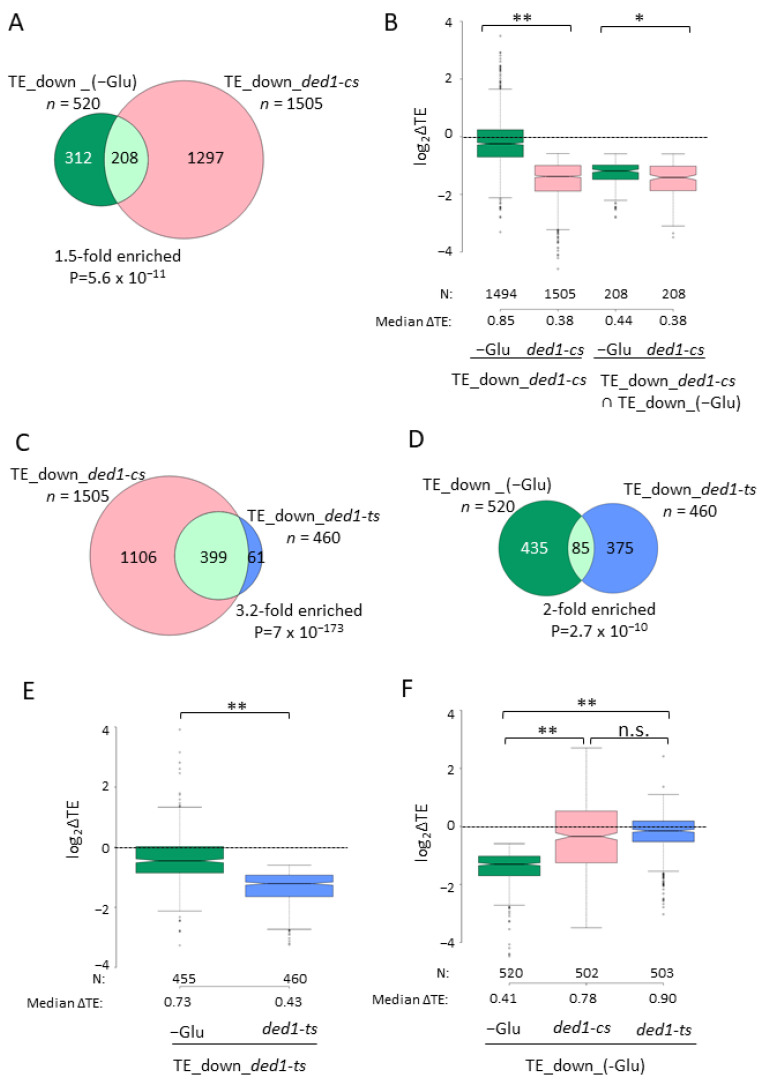
A significant fraction of Ded1-hyperdependent mRNAs in glucose-replete conditions is translationally down-regulated by glucose starvation. (**A**) Venn diagram of overlap between 520 mRNAs exhibiting ≥1.5-fold decrease in TE at FDR < 0.05 in WT cells under −Glu versus +Glu conditions (TE_down_(−Glu) group) and 1505 mRNAs showing a comparable decrease in TE in *ded1-cs* versus WT cells shifted to low temperature in a glucose-replete medium (TE_down_*ded1-cs* group) out of a total of 5474 mRNAs for which ribosome-profiling data were obtained in the *ded1-cs* experiment. TE change values were calculated from published ribosome-profiling data deposited on GEO: −Glu/+Glu, GSE51532; *ded1-cs*/WT, GSE 111255. For this and all Venn diagrams below, the fold-enrichment and *p* value for the overlap was assigned using a hypergeometric distribution. (**B**) Notched box plot analysis of the log2 fold-changes in TE (log2∆TE) observed in response to glucose starvation (−Glu), the *ded1-cs* mutation for the 1505 TE_down_*ded1-cs* mRNAs (col. 1–2), or the 208 mRNAs in the overlap between the TE_down_(−Glu) and TE_down_*ded1-cs* mRNAs in panel A (cols. 3–4). In this and all box plots below, each box depicts the interquartile range containing 50% of the data, intersected by the median; the notch indicates a 95% confidence interval (CI) around the median. Thus, if the notches in two boxes do not overlap, their medians differ with 95% confidence. If the notches of a box overlap zero on the *y*-axis, then the median log2FC for that group does not differ significantly from the median log2FC for all mRNAs, which equals zero due to normalization. *p* values from a Mann–Whitney U-test reporting the statistical significance of the difference in medians between the distributions in boxplots are indicated. Unlogged median TE change values are indicated below the *x*-axis. (**C**) Overlap between the mRNAs in the TE_down_*ded1-cs* group from (**A**) and the 460 mRNAs exhibiting a ≥1.5-fold decrease in TE at FDR < 0.05 in *ded1-ts* versus WT cells shifted to high temperature in a glucose-replete medium (TE_down_*ded1-ts* group) out of a total of 5494 mRNAs for which ribosome-profiling data were obtained in the *ded1-ts* experiment. (**D**) Analysis as in (**A**) but for the TE_down_(−Glu) and TE_down_*ded1-ts* groups of mRNAs from (**A**–**C**). (**E**) Notched box plots showing log2 fold-changes in TE in response to (−Glu) or *ded1-ts* for the 460 TE_down_*ded1-ts* mRNAs. (**F**) Notched box plots showing log2 fold-changes in TE in response to (−Glu), *ded1-cs*, or *ded1-ts* for the 520 TE_down_(−Glu) mRNAs. *p*-values computed from Mann–Whitney U-test are given (* *p* < 10^−3^; ** *p* < 10^−15^; n.s., not significant).

To assess the possible impact of the different strain backgrounds and culture conditions represented among the various analyzed ribosome-profiling datasets, we examined the correlations between the relative translation levels (ribosome-protected mRNA fragments (RPFs) summed over the coding sequences) for each of the 1566 Ded1 hyperdependent mRNAs (defined in Figure 1C), obtained from the ribosome-profiling results for the non-stressed WT control strains grown on glucose-containing medium at 30 °C for each of the four conditions of glucose starvation at 30 °C, heat-shock at 42 °C vs. 30 °C, rapamycin treatment at 30 °C, or the *ded1-ts* mutation at 37 °C. Correlation analyses of the RPFs for all 1542 Ded1 hyperdependent mRNAs (for which data were available for all four conditions) were conducted for all six pairwise comparisons. The Pearson correlation coefficients (*r*) ranged from 0.942 to 0.965, with *p* values of essentially zero, for all 6 comparisons (Supplementary Material File S1), indicating that the relative translation levels of the majority of Ded1-hyperdependent mRNAs were highly similar among the non-stressed WT controls for these three stress conditions and the *ded1-ts* mutation.

The hierarchical cluster analysis of TE changes was conducted with the R heatmap.2 function from the R ‘gplots’ library using the default hclust hierarchical clustering algorithm. For all notched box plots, constructed using a web-based tool at http://shiny.chemgrid.org/boxplotr/ (accessed on 21 November 2021), the upper and lower boxes contain the second and third quartiles and the band provides the median. If the notches in two plots do not overlap, there is roughly 95% confidence that their medians are different. The Mann–Whitney U-test was used to compute the statistical significance of the difference in medians between the distributions in boxplots. The significance of gene set overlaps in Venn diagrams was evaluated with the hypergeometric distribution by Fisher’s exact test.

## 3. Results

### 3.1. A Sizable Fraction of the mRNAs Whose Translation Is Most Strongly Down-Regulated by Glucose Starvation Are Ded1-Hyperdependent mRNAs

We first interrogated ribosome-profiling data from experiments in which WT yeast strain Σ1278b was cultured in a rich medium containing 2% glucose and then transferred to a medium lacking glucose and incubated for 3 h (dubbed −Glu) [27] (Table S1). It was reported that bulk polysome assembly was rapidly impaired within only 10 min of glucose withdrawal and partial recovery after 3 h, during which a broad reprogramming of translation occurred compared to glucose-replete cells (+Glu), as determined by the ribosome profiling of cells in the two conditions. Ribosome footprint profiling entails the deep-sequencing of RPFs in parallel with the RNA-Seq analysis of total mRNA. The ratio of sequencing reads of RPFs to mRNA for each gene provides a measure of TE. Because both RPF and mRNA reads for each gene are generally normalized to the total reads for that strain, the calculated TEs represent relative rather than absolute values; however, for brevity, we refer to them below simply as TE values. By employing DESeq2 analysis [25] to identify differentially translated mRNAs, we identified 520 mRNAs with a ≥1.5-fold decrease in TE in the −Glu versus +Glu datasets at an FDR of <0.05 (designated as TE_down_(−Glu) mRNAs).

Similarly analyzing our own ribosome-profiling data for the *ded1-cs* mutant cultured in a synthetic complete glucose-replete medium (SC) and shifted to a non-permissive temperature (15 °C) for 10 min, in parallel with isogenic WT *DED1* cells of strain BY4741 [5], allowed us to identify 1505 mRNAs showing a ≥1.5-fold decrease in TE in *ded1-cs* versus WT cells at FDR < 0.05 (designated TE_down_*ded1-cs* mRNAs), referred to below as Ded1-hyperdependent mRNAs to signify their greater than average requirement for Ded1 for efficient translation. There was a modest but statistically significant ~1.5-fold enrichment of the TE_down_(−Glu) mRNAs for these Ded1-hyperdependent mRNAs, with ~40% of the mRNAs translationally impaired on glucose starvation corresponding to ~27% of those impaired by *ded1-cs* (Figure 1A). (In all figures, results are consistently color-coded according to the stress condition or *ded1* mutation being examined.) The gene ontology (GO) analysis of the 208 mRNAs common to both sets (FunSpec at http://funspec.med.utoronto.ca/ (accessed on 21 November 2021)) showed significant enrichment for the biological functions of membrane transport and endocytosis (P_adj_ value (adjusted for Bonferroni correction) of <0.01). As a group, the Ded1-hyperdependent mRNAs show a significant, modest reduction in TE in −Glu conditions (median fold-change (FC) of 0.85) compared to the considerably larger effect of *ded1-cs* (median FC of 0.38) on these same mRNAs (Figure 1B, cols. 1–2). (In all box plots, when notches do not overlap between adjacent boxes, their medians differ with 95% confidence, and when notches do not overlap 0 in log(2) plots, the median for that group differs significantly from that for all mRNAs, which is unity). As expected, the group of 208 mRNAs in the overlap between the two groups exhibited a stronger TE reduction in −Glu (FC of 0.44), similar to that given by *ded1-cs* for these mRNAs (FC of 0.38) (Figure 1B, cols. 3–4). The relatively small median FC in TE of 0.85 observed in −Glu for all 1505 Ded1-hyperdependent mRNAs supports the finding in Figure 1A that only 14% of these 1505 mRNAs are down-regulated in −Glu by ≥1.5-fold.

We carried out the same analysis using ribosome-profiling data obtained for a *ded1-ts* mutant cultured in an SC medium and shifted to 37 °C for 2 h to inactivate the mutant Ded1 protein [5]. As noted previously, there was strong overlap (>3-fold enrichment) between the mRNAs whose TEs are down-regulated by ≥1.5-fold at FDR < 0.05 by the *ded1-ts* or *ded1-cs* mutations, with ~87% of the mRNAs affected by *ded1-ts* being similarly impaired by *ded1-cs,* even though these mutations were examined at the different temperatures of 37 °C versus 15 °C (Figure 1C). Thus, the cohort of Ded1-hyperdependent mRNAs is collectively well-defined by these two profiling experiments. The relatively greater number of Ded1-hyperdependent mRNAs identified in the *ded1-cs* mutant likely reflects its greater impact on translation initiation compared to *ded1-ts* [10], but it could also result from a greater requirement for Ded1 helicase function at low temperatures where RNA structures should be more stable. The group of 460 Ded1-hyperdependent mRNAs identified in *ded1-ts* cells again showed a moderate ~1.9-fold enrichment for mRNAs showing TE reductions in −Glu (Figure 1D) and exhibited a smaller median TE reduction in −Glu (FC of 0.73) compared to that given by the *ded1-ts* mutation (FC of 0.43; Figure 1E). Considering the 520 mRNAs in the group of TE_down_(−Glu), the *ded1-ts* and *ded1-cs* mutations conferred only modest median TE reductions of 0.90 and 0.78, respectively, compared to the larger median TE reduction of 0.41 provoked by glucose starvation for these mRNAs (Figure 1F).

Although we found that significant proportions of Ded1-hyperdependent mRNAs were translationally repressed in −Glu conditions, the observations that the majority of mRNAs repressed in this condition were not Ded1-hyperdependent mRNAs and that the Ded1-hyperdependent mRNAs exhibited a considerably smaller TE reduction during glucose starvation compared to that observed for the entire group of TE_down_(−Glu) mRNAs, suggesting that one or more other inhibitory mechanisms plays an equal or greater role than the inhibition of Ded1 function in reducing translation. The results also suggest that the reduction in Ded1 function in −Glu conditions was considerably smaller than that given by the *ded1-cs* and *ded1-ts* mutations in non-starved cells, as the resulting TE reductions for most Ded1-hyperdependent mRNAs in the −Glu conditions generally did not reach the threshold value of 1.5-fold and FDR < 0.05. However, because TE changes are determined relative to the average mRNA, the reduction in Ded1 function could be greater than that indicated by the TE reductions of the Ded1-hyperdependent mRNAs in −Glu conditions due to a reduction in the average TE conferred by other negative regulatory mechanisms at work in the starved cells. This underestimate of Ded1 inhibition would be appreciable if Ded1-hyperdependent mRNAs are generally immune to such other repressive mechanisms, which seems unlikely, or if the impact of reducing Ded1 function on Ded1-hyperdependent mRNAs was masked somehow by the other repressive mechanisms operating in glucose-starved cells. This caveat also applies to other stress conditions examined below.

We considered next the possibility that the broad reprogramming of TEs after 3 h of glucose starvation obscured a more pervasive down-regulation of Ded1-dependent mRNAs that occurs immediately following the shift to −Glu conditions. To address this, we examined ribosome-profiling data obtained after shifting WT strain BY4741 to an SC medium lacking glucose for only 15 min [28]. In addition to the expected collapse of bulk polysomes, it was reported that the expression levels and TEs of numerous heat-shock mRNAs were induced by this starvation regime. As our DESeq2 analysis of these data (Table S1) revealed only 16 mRNAs with significant TE reductions, we instead employed Xtail [26] for differential expression analysis to ensure maximum sensitivity and thereby identified 57 mRNAs with significantly reduced TEs (−Glu_O’Shea). However, these mRNAs showed slightly increased rather than decreased TEs in response to the *ded1-ts* and *ded1-cs* mutations in non-starved cells (Figure S1A, cols. 2–3), indicating that most of them are not Ded1-hyperdependent mRNAs. Furthermore, the groups of Ded1-hyperdependent mRNAs identified in *ded1-ts* or *ded1-cs* cells showed either a small increase or no change in median TE after 15 min of Glu starvation, in contrast to the 0.73- or 0.85-fold decreases conferred by 3 h of glucose deprivation in the −Glu dataset described above [27] (Figure S1B,C, cols. 2–3). These findings suggest that the rapid TE changes produced immediately after the withdrawal of glucose are not dominated by a decrease in Ded1 function. It is possible that because the vast majority of mRNAs are lost from polysomes immediately after glucose withdrawal, so it becomes difficult to discern differences in relative translational inhibition that become apparent after 3 h of glucose deprivation where translation has resumed to some extent and the reprogramming of relative initiation frequencies has occurred.

### 3.2. Translation of Very Few Ded1-Hyperdependent mRNAs Is Strongly Down-Regulated by Rapamycin Treatment

We next interrogated ribosome-profiling data from a study in which WT BY4741 cells growing in a rich (YPD) medium were treated with 12.5 nM rapamycin for 30 min to inhibit the TORC1 complex [29] (Table S1). Of the 147 mRNAs showing a ≥1.5-fold decrease in TE on Rap treatment at FDR < 0.05 (TE_down_Rap), we observed no significant enrichment for the Ded1-hyperdependent mRNAs identified in *ded1-cs* or *ded1-ts* cells (Figure S2A,B), and the latter Ded1-hyperdependent mRNAs showed little or no TE reductions on Rap treatment (Figure S2C, cols. 1 and 3). The *ded1-cs,* but not the *ded1-ts,* mutation conferred a modest TE reduction of 0.86-fold for the 147 mRNAs showing Rap-induced TE reductions (Figure S2D, cols. 2–3). Overall, the TEs of the vast majority (97–98%) of Ded1-hyperdependent mRNAs were not found to be appreciably down-regulated by Rap treatment, suggesting that Ded1 function is impaired by the Rap inhibition of TORC1 to a much smaller degree than by the *ded1* mutations. Moreover, the mRNAs that are translationally repressed the most by Rap generally do not exhibit a heightened dependence on Ded1 in non-treated cells.

### 3.3. Only a Small Fraction of the mRNAs Whose Translation Is Most Strongly Down-Regulated by a Shift from 40 °C to 42 °C Is Ded1-Hyperdependent

To determine whether Ded1-hyperdependent mRNAs are translationally impaired by heat-shock, we first examined ribosomal-profiling data from a study in which WT cells in the W303 background were grown in a rich (YPD) medium at 30 °C and then incubated for 10 min at 30, 40, or 42 °C [19] (Table S1). Noting that gene expression changes predominantly occurred at the translational level between the conditions of 42 and 40 °C [19], the authors primarily focused on the TE changes observed between these two elevated temperatures. Our DESeq2 analysis identified 258 mRNAs with a ≥1.5-fold decrease in TE following a shift to 42 °C versus a shift to 40 °C at FDR < 0.05 (TE_down_42C_v_40C). These mRNAs showed a ~1.9-fold enrichment for Ded1-hyperdependent mRNAs identified in *ded1-ts* cells (Figure S3B), but no significant enrichment for the Ded1-hyperdependent mRNAs identified in *ded1-cs* cells (Figure S3A). Moreover, they showed no significant change in median TE conferred by either *ded1* mutation (Figure S3D, cols. 3–4). The two Ded1-hyperdependent groups of mRNAs likewise showed only small reductions in median TE from 0.97- to 0.88-fold in the shift to 42 °C versus 40 °C (Figure S3C, cols. 1 and 3), indicating that Ded1 function might be impaired by heat-shock at 42 °C versus 40 °C to a much smaller degree than that conferred by the *ded1-cs* and *ded1-ts* mutations in non-heat-shocked cells. Our findings that most of the mRNAs translationally impaired by heat-shock at 42 °C versus 40 °C are not hyperdependent on Ded1 in non-shocked cells (Figure S3A,B,D) seems to be at odds with the conclusion that the sequestration of Ded1 is a major driver of TE changes occurring between these two heat-shock conditions [19].

### 3.4. Many Ded1-Hyperdependent mRNAs Are Translationally Down-Regulated by the DED1-IDRm Mutation That Increases Ded1 Condensation at 42 °C

**Figure 2:**
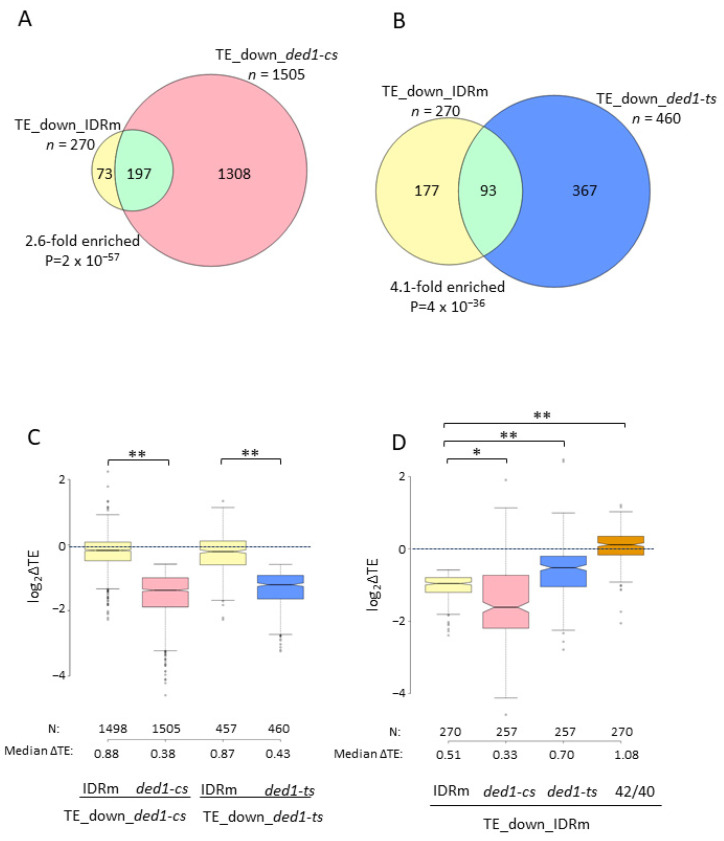
A significant fraction of Ded1-hyperdependent mRNAs translationally down-regulated by the *ded1-ts* or *ded1-cs* mutations is translationally down-regulated by the *DED1-IDRm* mutation at 42 °C. (**A**,**B**) Overlap between 270 mRNAs exhibiting ≥1.5-fold reductions in TE in *DED1-IDRm* versus WT cells at 42 °C (TE__down_IDRm) and either the TE_down_*ded1-cs* group from Figure 1A (**A**) or the TE_down_*ded1-ts* mRNAs from Figure 1C (**B**). (**C**) Notched box plots showing log_2_ fold-changes in TE in response to the *ded1-IDRm* mutation at 42 °C (*IDRm*/WT), *ded1-cs,* or *ded1-ts* mutations for the 1505 TE_down_*ded1-cs* mRNAs (cols. 1–2) or 460 TE_down_*ded1-ts* mRNAs (cols. 3–4). (**D**) Log_2_ fold-changes in TE in response to the *ded1-IDRm* mutation at 42 °C, *ded1-cs, ded1-ts,* or heat-shock at 42 °C vs. 40 °C for the 270 TE_down_IDRm mRNAs. One outlier mRNA was omitted from the plot to expand the *y*-axis scale. *p*-values computed from Mann–Whitney *U*-test are given (* *p* < 10^−10^; ** *p* < 10^−15^).

In the same study on the effects of heat-shock on translation, Iserman et al. described the Ded1-IDRm variant harboring substitutions of a low-complexity sequence in the NTD, which forms insoluble condensates at a lower temperature compared to WT Ded1, particularly at 42 °C [19]. Their ribosome profiling of *DED1-IDRm* cells after a 10 min shift to 42 °C indicated that the group of mRNAs translationally repressed in comparison to temperature-shifted WT cells (TE_down_IDRm) are enriched for longer 5′UTRs with a greater potential for secondary structure, consistent with a reduction in Ded1 function resulting from the enhanced aggregation of the Ded1-IDRm variant [19]. Supporting this interpretation, we found substantial overlap between the 270 TE_down_IDRm mRNAs (identified by DESeq2 with a ≥1.5-fold decrease in TE at FDR < 0.05) and the larger groups of mRNAs translationally down-regulated in *ded1-cs* or *ded1-ts* cells (Figure 2A,B). Furthermore, the TE_down_IDRm group exhibited median TE reductions of 0.33- and 0.70-fold in the *ded1-ts* and *ded1-cs* mutants, respectively, similar in degree to the reduction of 0.52-fold conferred by the *DED1-IDRm* mutation after a 42 °C heat-shock (Figure 2D, cols. 1–3). (These mRNAs showed small increases rather than decreases in TE on the heat-shock of WT cells at 42 °C versus 40 °C (Figure 2D, col. 4), further suggesting that the mRNAs translationally down-regulated in WT cells at 42 °C versus 40 °C are not primarily affected by the loss of Ded1 function under these heat-shock conditions.) It is noteworthy, however, that the *DED1-IDRm* mutation produced median TE reductions for the two groups of Ded1-hyperdependent mRNAs that were considerably smaller than those conferred by the *ded1-cs* and *ded1-ts* mutations (Figure 2C, cols. 1 and 3 vs. 2 and 4), suggesting a smaller reduction in Ded1 activity by the 42 °C heat-shock of *DED1-IDRm* cells versus the *ded1-cs* or *ded1-ts* mutations at their restrictive temperatures. One explanation for the fact that all three *DED1* mutations conferred similar TE reductions for the TE_down_IDRm group of mRNAs (Figure 2D, cols. 1–3) could be that this subset of Ded1-hyperdependent mRNAs is preferentially sequestered in condensates with the Ded1-IDRm variant in addition to being translationally impaired by the reduction in soluble Ded1 protein in the cytoplasm. In summary, the results in Figure 2 support the conclusion that the *DED1-IDRm* mutation reduces Ded1 function on heat-shock through enhanced Ded1 condensation, which reduces the TEs of a substantial number of Ded1-hyperdependent mRNAs [19].

### 3.5. A Sizable Fraction of Ded1-Hyperdependent mRNAs Is Translationally Down-Regulated by a Shift from 30 °C to 42 °C

**Figure 3:**
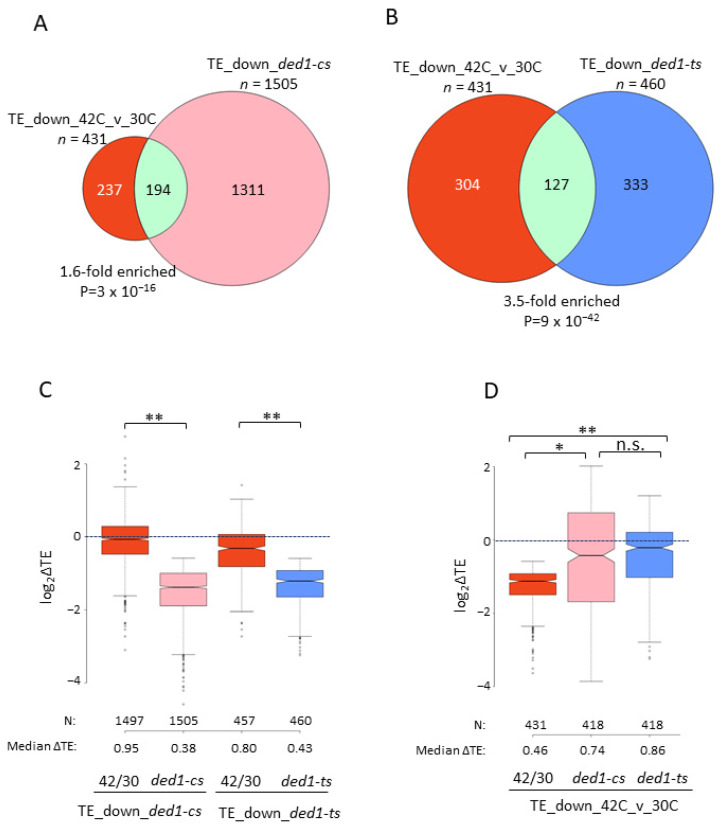
A significant fraction of Ded1-hyperdependent mRNAs translationally down-regulated by the *ded1-ts* or *ded1-cs* mutations is translationally down-regulated by the heat-shock of WT cells at 42 °C versus 30 °C. (**A**,**B**) Overlap between the 431 TE_down_42C_v_30C mRNAs and either the TE_down_*ded-cs* (**A**) or TE_down_*ded-ts* (**B**) mRNAs described in Figure 1. (**C**) Log_2_ fold changes in TE in response to the heat-shock of WT cells at 42 °C versus 30 °C (42C/30C), *ded1-cs,* or *ded1-ts* for the 1505 TE_down_*ded1-cs* mRNAs (cols. 1–2) or 460 TE_down_*ded1-ts* mRNAs (cols. 3–4). (**D**) Log_2_ fold-changes in TE in response to heat-shock at 42 °C vs. 30 °C, *ded1-cs,* or *ded1-ts* for the 431 TE_down_42C_v_30C mRNAs. One outlier mRNA was omitted from the box plots in C and D to expand the *y*-axis scale. *p*-values computed from Mann–Whitney *U*-test are given (* *p* < 10^−13^; ** *p* < 10^−15^; n.s., not significant).

In view of our findings above supporting the conclusion that *DED1-IDRm* impairs Ded1 function at 42 °C, we re-evaluated the data from Iserman et al. (2020) to determine whether mRNAs exhibiting TE changes conferred by heat-shock at 42 °C compared to unstressed cells at 30 °C are enriched for Ded1-hyperdependent mRNAs. Indeed, a group of 431 TE_down_42C_v_30C mRNAs identified via the DESeq2 analysis of the published data (Table S1) was significantly enriched for Ded1-hyperdependent mRNAs defined by the *ded1-cs/ded1-ts* mutations (Figure 3A,B) and also showed significant median TE reductions in response to these two mutations (Figure 3D, cols. 3–4). Moreover, heat-shock at 42 °C versus 30 °C conferred a TE reduction of 0.80-fold for the Ded1-hyperdependent mRNAs identified by *ded1-ts* (Figure 3C, col. 3). Overall, these results support the notion that Ded1 function is reduced by a heat-shock of 42° in comparison to non-stressed cells, which could involve the condensation of Ded1 at the elevated temperature [19].

### 3.6. TE Changes Conferred by ded1-ts Most Closely Resemble Those Conferred by Glucose Starvation and Heat-Shock at 42 °C versus 30 °C

**Figure 4:**
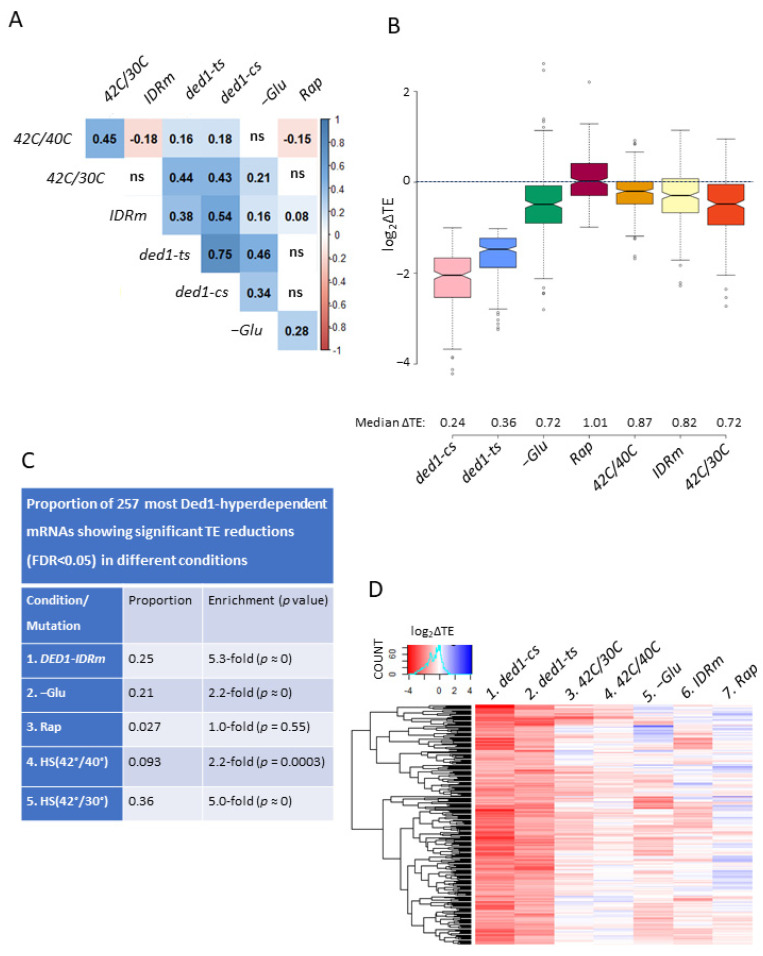
The TE changes produced by the *DED1-IDRm* mutation at 42 °C, glucose starvation, and heat-shock at 42 °C versus 30 °C most closely resemble those conferred by the *ded1-ts* and *ded1-cs* mutations. (**A**) Spearman correlation coefficients of TE changes for 745 Ded1-dependent mRNAs exhibiting ≥1.5-fold increases or decreases in TE in *ded1-ts* versus WT cells at FDR < 0.05 in response to the indicated mutations or stresses. ns, correlations not statistically significant. (**B**) Notched box plots showing log_2_ fold-changes in TE in response to the indicated mutations or stresses for the group of 257 mRNAs showing >2-fold reductions in TE in both *ded1-ts* and *ded1-cs* mutants (2X_TE_down_*ded1-cs*_∩_*ded1-ts*). (**C**) Overlap analyses of the 257 Ded1-hyperdependent mRNAs described in (**B**) and the groups of mRNAs showing significant TE reductions in response to the indicated *ded1* mutations or stresses. (**D**) Hierarchical cluster analysis results of TE change values for the 257 mRNAs in (**B**) in response to the indicated *ded1* mutations or stresses; this analysis was conducted with the R heatmap.2 function from the R ‘gplots’ library using the default hclust hierarchical clustering algorithm.

To explore which of the stress conditions examined above most closely resembles the mutational impairment of Ded1 function, we first computed the Spearman rank correlation coefficients for the TE changes conferred by the different conditions for the group of 745 mRNAs exhibiting significant TE increases or decreases of >1.5-fold (at FDR < 0.05) in response to the *ded1-ts* mutation. As expected, the strongest correlation was observed for the TE changes conferred for these mRNAs by the *ded1-cs* mutation (ρ = 0.75), and a good correlation was also observed for the *DED1-IDRm* mutation at 42 °C (ρ = 0.38) (Figure 4A). Among the stress conditions, the TE changes conferred by glucose starvation and heat-shock at 42 °C versus 30 °C showed stronger correlations (ρ = 0.46 and ρ = 0.44, respectively) than those produced by heat-shock at 42 °C versus 40 °C (ρ = 0.16), and those conferred by Rap treatment showed no significant correlation (*p* > 0.05) with the TE changes evoked by *ded1-ts*. The TE changes conferred by the *ded1-cs* mutation also showed strong correlations with those evoked by *DED1-IDRm* (ρ = 0.54), heat-shock at 42 °C versus 30 °C (ρ = 0.43), and and glucose starvation (ρ = 0.34) for these Ded1-hyperdependent mRNAs.

A similar picture emerged when considering the TE changes produced in the three stress conditions for a select group of 258 mRNAs that exhibited TE reductions of >2-fold (FDR < 0.05) in both *ded1-cs* and *ded1-ts* mutants, representing the mRNAs most strongly dependent on Ded1 at both high and low growth temperatures. Compared to the strong median TE reductions of 0.24- and 0.36-fold for these mRNAs in the *ded1-cs* and *ded1-ts* mutants, respectively, glucose starvation, heat-shock at 42 °C versus 30 °C, and the *DED1-IDRm* mutation produced the next largest fold-reductions of 0.72, 0.72, and 0.81, respectively, followed by 0.88 under heat-shock at 42 °C versus 40 °C. In contrast, Rap treatment conferred no significant change in TE for this cohort (Figure 4B). Consistent with these findings, these 258 Ded1-hyperdependent mRNAs presented highly significant enrichments for mRNAs, showing TE reductions in response to heat-shock at 42 °C versus 30 °C, the *DED1-IDRm* mutation, and glucose starvation but lesser or no significant enrichment for mRNAs affected by heat-shock at 42 °C vs. 40 °C or by Rap, respectively (Figure 4C). When the TE changes for each of the 258 Ded1-hyperdependent mRNAs defined above was visualized by a heat-map following the hierarchical clustering of the TE changes (Figure 4D), it was again apparent that the TE changes conferred by the *ded1-ts* and *ded1-cs* mutations (cols. 1–2) were most congruent in terms of number and magnitude of changes to those produced by −Glu, heat-shock at 42 °C versus 30 °C, and the *DED1-IDRm* mutation (cols. 3, 5–6). The fact that glucose starvation conferred increased rather than decreased TEs for a subset of these Ded1-hyperdependent mRNAs (blue lines at top of col. 5) might have resulted from the regulation of other factors in addition to Ded1 that could counteract the consequences of reduced Ded1 function under these conditions. Many of the mRNAs did show TE reductions upon heat-shock at 42 °C versus 40 °C (col. 4), but they were generally smaller in magnitude than those for heat-shock at 42 °C versus 30 °C and −Glu. Rap treatment showed the fewest similarities in TE changes to the *ded1* mutations (col. 7 vs. 1–2).

### 3.7. Evidence That Translational Impairment of a Subset of Ded1-Hyperdependent mRNAs during Stress Involves Compound Reductions in Ded1, eIF4B, and eIF4A Functions

As noted above, recent evidence indicates that the withdrawal of glucose elicits the rapid dissociation of Ded1 and initiation factors eIF4A and eIF4B from the translation initiation regions of many mRNAs, as well as a more gradual release upon heat-shock [20]. Hence, we wondered whether the subset of Ded1-hyperdependent mRNAs that are translationally down-regulated the most by these stresses might also exhibit a heightened dependence on eIF4A or eIF4B that exceeds that seen for other Ded1-hyperdependent mRNAs. If so, the coupled dissociation of eIF4A or eIF4B with Ded1 might confer independent reductions in the initiation rate that collectively confer the marked TE reductions observed for this subset of Ded1-hyperdependent mRNAs upon glucose starvation or heat-shock.

**Figure 5:**
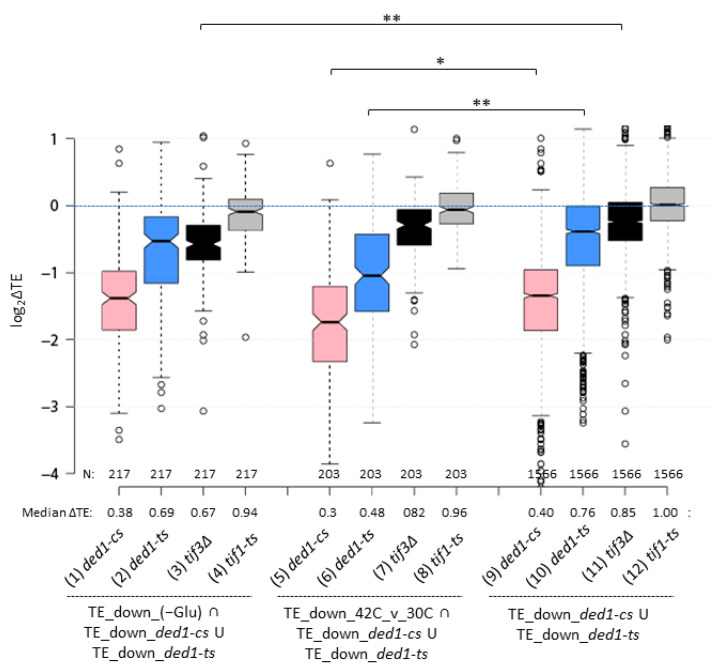
Elimination of eIF4B or inactivation of eIF4A preferentially reduces the TEs of a subset of Ded1-hyperdependent mRNAs down-regulated by glucose starvation. Log_2_ fold-changes in TE in response to the *ded1-cs*, *ded1-ts*, and either *tif3Δ* or *tif1-ts* at 37 °C for the 1566 mRNAs showing 1.5-fold TE reductions in response to either *ded1-cs* or *ded1-ts* mutations (cols. 9–12) or the subsets of these mRNAs that also show TE reductions in response to either glucose starvation (217 mRNAs in cols. 1–4) or heat-shock at 42 °C versus 30 °C (203 mRNAs in cols. 5–8). *p*-values computed from Mann–Whitney *U*-test are given (* *p* < 0.05; ** *p* < 10^−4^).

To explore this concept for glucose starvation, we first identified a group of 217 Ded1-hyperdependent mRNAs, among all 1566 TE_down mRNAs identified in either the *ded1-ts* or *ded1-cs* mutants, that displayed TE reductions upon 3 h of glucose starvation in the profiling data analyzed above [27] (Table S1). We then asked whether these 217 mRNAs displayed TE reductions in our previous analyses of a *tif3Δ* mutant lacking eIF4B [30] or a temperature-sensitive eIF4A mutant, *tif1-ts* [10], in a glucose-replete medium. Interestingly, the 217 Ded1-hyperdependent mRNAs down-regulated in −Glu showed a marked TE reduction in response to *tif3Δ* comparable in degree to that conferred by the *ded1-ts* mutation (Figure 5, cols. 1–3), which was significantly greater than the TE reduction conferred by *tif3Δ* for the entire group of 1566 Ded1-hyperdependent mRNAs (col. 3 vs. 11). A qualitatively similar result was obtained for the *tif1-ts* mutation at 37 °C (Figure 5, col. 4 vs. 12); however, the magnitude of the TE reduction given by *tif1-ts* was smaller than that conferred by *tif3Δ* for the Ded1-hyperdependent subset translationally down-regulated in −Glu conditions (Figure 5, cols. 3–4). As shown previously [10], the *tif1-ts* mutation alters the relative TEs of very few mRNAs even though bulk translation is markedly reduced because the translation of most mRNAs is lowered by similar amounts to produce a change in relative TE of ≈1.0, as seen here for all Ded1-hyperdependent mRNAs (Figure 5, col. 12). Overall, these findings support the idea that the subset of Ded1-hyperdependent mRNAs translationally impaired during glucose limitation exhibit a compound reduction in Ded1, eIF4B, and eIF4A functions.

We performed the same analyses of the effects of *tif3Δ* and *tif1-ts* mutations for the subset of 203 Ded1-hyperdependent mRNAs that are translationally down-regulated by heat-shock at 42 °C versus 30 °C. Similar findings emerged for *tif1-ts,* which conferred a small reduction in median TE for this subset compared to no change observed for the entire group of Ded1-hyperdependent mRNAs (Figure 5, col. 8 vs. 12). In contrast, the relatively greater TE reductions conferred by *tif3Δ* were similar in magnitude for this subset and the group of all Ded1-hyperdependent mRNAs (Figure 5, col. 7 vs. 11). Thus, the Ded1-hyperdependent mRNAs translationally impaired during heat-shock did not show a heightened dependence on eIF4B compared to other Ded1-hyperdependent mRNAs. However, this subset did show a relatively greater reduction in TE conferred by the *ded1-ts* and *ded1-cs* mutations, indicating a greater dependence on Ded1 compared to other Ded1-hyperdependent mRNAs (Figure 5, cols. 5–6 vs. 9–10). Thus, the results support the possibility that the subset of Ded1-hyperdependent mRNAs that are translationally impaired by heat-shock at 42 °C versus 30 °C undergo greater than average reductions in both Ded1 and eIF4A function compared to other Ded1-hyperdependent mRNAs.

Examining the ribosome-profiling data from Zinshteyn et al. [31] (Table S1) regarding the depletion of eIF4G1 on a galactose medium in a strain lacking its paralog eIF4G2 in the same way provided no evidence that the subsets of Ded1-hyperdependent mRNAs showing TE reductions in response to −Glu, *DED1-IDRm*, or heat-shock at 42° versus 30 °C are unusually dependent on eIF4G (Figure S4A, cols. 1–3 vs. 4). Furthermore, the mRNAs showing TE reductions upon Rap treatment identified above (TE_down_Rap) did not exhibit a heightened dependence on eIF4G for translation in non-treated cells (Figure S4B, col. 2), suggesting that the degradation of eIF4G triggered by the inhibition of TORC1 [22,23] is not a major driver of the specific TE reductions conferred by Rap treatment.

## 4. Discussion

We made use of the ribosome-profiling analyses of *ded1* mutations that impair Ded1 helicase activity [5,10] to define a group of mRNAs unusually dependent on Ded1 for translation in order to evaluate whether WT Ded1 function, and thus the translation of these Ded1-hyperdependent mRNAs, is reduced during different stresses. In our previous studies, we examined cold-sensitive or heat-sensitive *ded1* mutants after the shortest incubations at their non-permissive temperatures sufficient to substantially reduce bulk translation in an effort to minimize the indirect effects of impairing Ded1 function. Interrogating recent results from measuring Ded1 binding to yeast mRNAs in vivo by UV crosslinking [20] revealed that the group of 1566 mRNAs judged to be Ded1-hyperdependent via the ribosome profiling of the *ded1-cs* and *ded1-ts* mutants exhibited significantly greater Ded1 occupancies in their 5′UTRs compared to all yeast mRNAs that showed detectable binding, whereas most of the corresponding group of 1755 mRNAs judged to be hypodependent on Ded1 by the same criteria (exhibiting increased not decreased relative TEs in *ded1* mutants) showed no detectable Ded1 binding and lower than average occupancies for the small proportion that does bind Ded1 (Figure S6). These findings further support the idea that Ded1 acts directly on the cohort of Ded1-hyperdependendent mRNAs to stimulate their translation initiation in vivo, which we succeeded in reconstituting for representative hyperdependent mRNAs in a purified system [11].

By interrogating published ribosome-profiling data obtained for glucose starvation, different regimes of heat-shock, the *DED1-IDRm* mutation, and the inhibition of TORC1 by rapamycin for the reductions in TE of Ded1-hyperdependent mRNAs, we found evidence consistent with a marked reduction in Ded1 function only for a subset of these conditions, and even for those, the degree of Ded1 impairment appeared to be less than that conferred by the *ded1-ts* or *ded1-cs* mutations affecting the Ded1 catalytic domain. Interestingly, for each condition, we observed that only a subset of all Ded1-hyperdependent mRNAs displayed significant TE reductions compared to unstressed control cells. In addition, many mRNAs translationally down-regulated by stress are not hyperdependent on Ded1, suggesting negative regulation of other factors. Indeed, we obtained evidence consistent with the idea that diminished Ded1 function during glucose starvation combines with the inhibition of eIF4B and eIF4A to evoke the marked reductions in translation observed for the particular subset of Ded1-hyperdependent mRNAs whose translation is down-regulated the most in glucose-starved cells.

The *DED1-IDRm* mutation confers a modest but significant reduction in the median TE, by ~0.87-fold, for the different groups of Ded1-hyperdependent mRNAs whose translation is impaired by *ded1-cs* or *ded1-ts*. As summarized in Table 1, of the 282 mRNAs showing TE reductions in response to *DED1-IDRm* at 42 °C, the majority (74%) also showed TE reductions in response to either *ded1-ts* or *ded1-cs* mutations (Table 1, row 1, col. 2), which were similar in magnitude to those conferred by *ded1-ts/ded1-cs* for all Ded1-hyperdependent mRNAs (Figure 2D, cols. 1–3). Thus, the TE_down_*IDRm* mRNAs are highly Ded1-hyperdependent. On the other hand, these 282 mRNAs comprised ≤25% of the larger groups of mRNAs showing TE reductions in response to *ded1-cs* or *ded1-ts* mutations, including the 258 mRNAs most heavily dependent on Ded1 (i.e., showing >2-fold TE reductions in both *ded1-cs* and *ded1-ts* mutants) (Table 1, row 1, cols. 3–5). Our findings that only a minority of Ded1-hyperdependent mRNAs were impaired by *DED1-IDRm* and that *DED1-IDRm* conferred a small median TE reduction compared to *ded1-ts* and *ded1-cs* for the entire group of Ded1-hyperdependent mRNAs (Figure 2C, cols. 1 and 3) might indicate that *DED1-IDRm* only moderately reduces Ded1 function at 42 °C compared to the *ded1-ts* and *ded1-cs* mutations at their restrictive temperatures. This seems unexpected considering that 80% of Ded1-IDRm were found to be insoluble at 42 °C compared to 50% of WT Ded1 [19]. Alternatively, the requirement for Ded1 could be diminished at 42 °C (where the *IDRm* mutation was examined) compared to the temperatures of 37 °C/15 °C used to inactivate *ded1-ts/ded1-cs,* e.g., by changes in mRNA structure or the expression or activities of other initiation factors under heat-shock conditions. An intriguing possibility is that the marked TE reductions observed for the TE_down_*IDRm* set of mRNAs reflect their preferential condensation with the Ded1-IDRm variant at 42 °C, thus reducing their proportions in the cytoplasmic pool [19], combined with the reduction in soluble Ded1-IDRm levels that result from its enhanced condensation. However, this possibility assumes that the mRNAs in Ded1 condensates are translationally repressed, whereas single molecule observations on mammalian cells have indicated that the fractions of reporter mRNAs found sequestered in SGs are translated at only slightly lower rates than the fractions that remains cytoplasmic [32]. It is also important to note that relatively small fractions of mRNAs are found to be sequestered in SGs in stressed cells [32,33,34], and it is currently unclear what proportions of Ded1-hyperdependent mRNAs become insoluble at 42 °C in cells expressing either WT Ded1 or the Ded1-IDRm variant.

Among the four stress conditions examined for WT cells, 3 h of glucose starvation and acute heat-shock at 42 °C versus 30 °C were found to most closely resemble the *ded1-cs* and *ded1-ts* mutations in down-regulating the translation of Ded1-hyperdependent mRNAs. Thus, nearly one-half of the mRNAs showing TE reductions on −Glu or HS (42°/30°) were also impaired by either *ded1-ts* or *ded1-cs* (Table 1, rows 2 and 5, col. 2) and 21–36% of the 258 most highly Ded1-hyperdependent mRNAs were down-regulated by −Glu or HS (42°/30°) (Table 1, rows 2 and 5, col. 5), which represent highly significant overlaps among these groups. These findings, together with the significant down-regulation of Ded1 targets by *DED1-IDRm* (noted above) and its known enhancement of Ded1 condensation [19], support the notion that Ded1 function is down-regulated during glucose starvation and heat-shock in a manner that is enhanced by Ded1 condensation. On the other hand, the −Glu and HS (42°/30°) stresses reduce the translation of <20% of the groups of Ded1-hyperdependent mRNAs (Table 1, rows 2 and 5, cols, 3–5), which might be explained by proposing a smaller reduction in Ded1 function on glucose starvation or heat-shock compared to *ded1-ts*/*ded1-ts* mutations or by positing other changes evoked by these stresses that reduce the requirement for Ded1 for the robust translation of its target mRNAs. The subset of Ded1-hyperdependent mRNAs showing TE reductions in response to HS (42°/30°) were found to be unusually hyperdependent on Ded1 (Figure 5, cols. 5–6 vs. 9–10) and thus might have been substantially impaired despite only a moderate reduction in Ded1 function upon heat-shock. However, this explanation would not apply to the Ded1-hyperdependent mRNAs translationally impaired by −Glu, which have shown average Ded1 dependence (Figure 5, cols. 1–2 vs. 9–10). As suggested above for the *DED1-IDRm* mutation, the subset of Ded1-hyperdependent mRNAs translationally impaired by −Glu or HS (42°/30°) might be especially prone to condensation and experience translational repression, at least in part, by exiting the cytoplasmic pool in Ded1 condensates.

**Figure 6:**
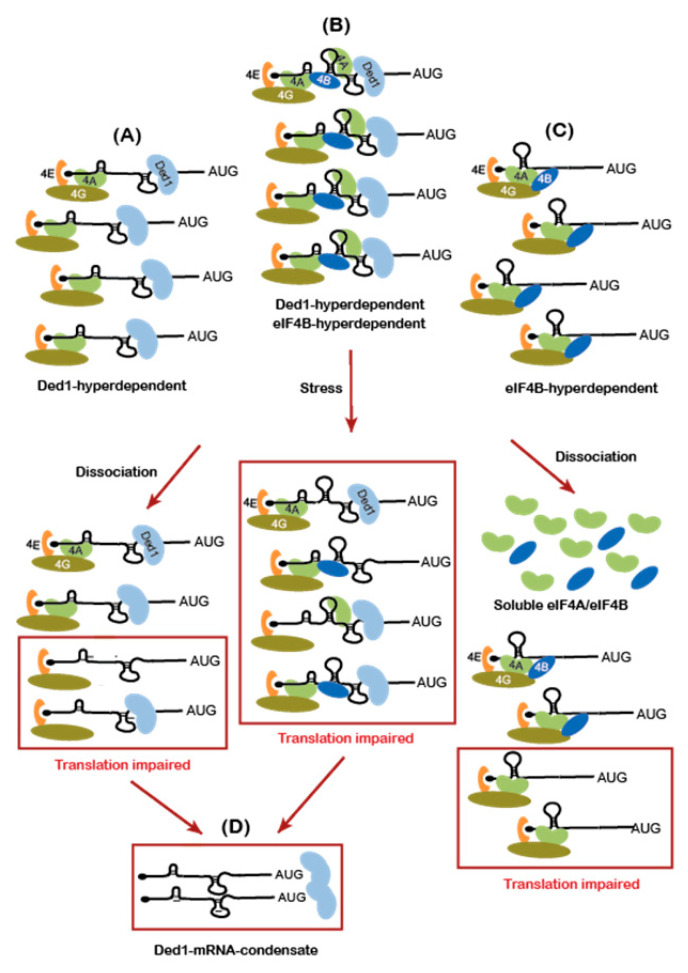
Hypothetical model for compound effects of the Ded1 condensation and dissociation of eIF4B and eIF4A from mRNAs in conferring the reduced translation of a select group of Ded1-hyperdependent mRNAs with a heightened dependence on eIF4B/eIF4A during glucose starvation. Three groups of mRNAs are depicted, two of which are hyperdependent on Ded1 (**A**,**B**) and two of which are hyperdependent on eIF4B (**B**,**C**). The Ded1-hyperdependent mRNAs have cap-distal stem-loop structures dependent on Ded1 for unwinding to enable the efficient scanning of the 5′UTR (**A**,**B**), and the group co-dependent on Ded1 and eIF4B (**B**) additionally requires eIF4B/eIF4A function for this process. The group dependent on eIF4B but not Ded1 (**C**) is pictured with cap-proximal structures that require eIF4A/eIF4B unwinding to facilitate PIC attachment to the mRNA 5′ end. It was observed that during glucose starvation, about twice as much of the bound eIF4A and eIF4B dissociated from the mRNA compared to that observed for Ded1 (Bresson et al. 2020). For simplicity, we depict the dissociation of 50% of the bound eIF4A/eIF4B and 25% of the bound Ded1 from all mRNAs bound by these factors, somewhat less than the ~66% and 33% experimentally measured for glucose starvation (Bresson et al. 2020). Owing to the compound effects of the dissociation of eIF4A, eIF4B, and Ded1, group (**B**) showed a greater proportion of mRNAs exhibiting translational reductions (red box), compared to the other two groups that had heightened dependence on only Ded1 (**A**) or eIF4B (**C**). It was also found that a subset of mRNAs that exhibited a loss of Ded1 from 5′UTRs maintained Ded1 bound to the coding sequences following glucose starvation. This subset of translationally inactive Ded1-mRNA complexes could lead to the formation of Ded1-mRNA condensates that remove a fraction of these mRNAs and a proportion of Ded1 from the cytoplasmic pool (**D**) that might decrease Ded1 function and the translation of Ded1-hyperdependent mRNAs in glucose-starved cells. For simplicity, the Ded1 bound 3′ of the AUG start codon (i.e., in coding sequences) was not depicted in (**A**,**C**) because this attribute only pertains to a subset of mRNAs. In heat-shocked cells, dissociation of Ded1 is more pronounced and on par with eIF4A/eIF4B dissociation (Bresson et al. 2020), which might explain why the subset of Ded1-hyperdependent mRNAs that are translationally impaired by heat-shock are not unusually dependent on eIF4B but rather more dependent on Ded1 itself compared to all Ded1-hyperdependent mRNAs, as shown above in Figure 5.

An intriguing alternative model to account for the subset of Ded1-hyperdependent mRNAs translationally impaired by −Glu or HS (42°/30°) is that they undergo compound reductions in their stimulation by Ded1, eIF4B, and eIF4A, all of which enhance mRNA recruitment or scanning. eIF4B and eIF4A were found to dissociate en masse from mRNAs following −Glu or heat-shock, in parallel with the selective dissociation of Ded1 from initiation regions of mRNAs [20]. We found that the subset of Ded1-hyperdependent mRNAs translationally impaired by −Glu showed a heightened dependence on eIF4B and a somewhat greater dependence on eIF4A compared to all Ded1-hyperdependent mRNAs (Figure 5, col. 3–4 vs. 11–12). Thus, the loss of eIF4B and eIF4A from most mRNAs in −Glu conditions could be expected to preferentially reduce the translation of this subset of Ded1-hyperdependent mRNAs down-regulated in −Glu conditions. This impairment, combined with the dissociation of Ded1 from initiation regions (and possibly its exit from the soluble pool by condensation) could produce additive reductions in TE that exceed those displayed by Ded1-hyperdependent mRNAs with only average dependence on eIF4B and eIF4A (Figure 6). The subset of Ded1-hyperdependent mRNAs showing TE reductions in HS (42°/30°) showed a greater than average dependence on eIF4A but not eIF4B (Figure 5, col. 7–8 vs. 11–12); however, as noted above, they were shown to have a heightened dependence on Ded1 itself. For this latter group of mRNAs, therefore, the dissociation of eIF4B, eIF4A, and Ded1 from initiation regions, as well as their condensation with Ded1, may produce additive reductions in TE greater than that experienced by the entire cohort of Ded1-hyperdependent mRNAs.

It is noteworthy that the *ded1-cs* and *ded1-ts* mutations produced only moderate TE reductions of between 0.74- and 0.9-fold for the entire TE_down_(−Glu) and TE_down_42C_v_30C groups of mRNAs (Figure 1F, cols. 2–3, and Figure 3D, cols. 2–3) and that large proportions of these two groups were not Ded1-hyperdependent mRNAs (Figure 1A,D and Figure 2A,B). These findings suggest that the reduced translation of many mRNAs under these stress conditions primarily results from the down-regulation of other factors besides Ded1, which likely include eIF4A and eIF4B [20,21].

Compared to the effects of the *DED1-IDRm* mutation and the −Glu and HS (42°/30°) stresses discussed above, a heat-shock of WT cells at 42° versus 40° translationally impaired a much smaller proportion of Ded1-hyperdependent mRNAs (Table 1, row 3, cols. 3–5), and less than one-third of the mRNAs translationally impaired by HS (42°/40°) were Ded1-hyperdependent (Table 1, row 3, col. 2). Moreover, the *ded1-ts* and *ded1-cs* mutations conferred no reduction in the median TE of the group of mRNAs down-regulated by HS (42°/40°) (Figure S3D). It is also noteworthy that the *DED1-IDRm* mutation at 42 °C and HS (42°/40°) did not confer correlated TE changes for Ded1-hyperdependent mRNAs (Figure 4A). Moreover, unlike the significant overlap of mRNAs impaired by *DED1-IDRm* and HS (42°/30°) (Figure S5B), there was no significant overlap in the groups of mRNAs showing TE reductions in these two conditions (Figure S5A). Hence, by all accounts, the TE reductions conferred by heat-shocking WT cells at 42 °C versus 40 °C do not appear to be significantly related to those given by *DED1-IDRm* and the *ded1-ts/ded1-cs* mutations. While our analyses are consistent with the possibility that enhanced condensation of the Ded1-IDRm variant at 42 °C and heat-shock of WT cells at 42 °C versus 30 °C both inhibit Ded1 function, they do not support the contention that the increased condensation of WT Ded1 at 42 °C versus 40 °C is associated with an appreciable reduction in Ded1 activity [19].

There is little indication from our analyses that down-regulating TORC1 with the Rap treatment employed by Nedialkova et al. [29] impairs Ded1 function. Only ~3% of all Ded1-hyperdependent mRNAs exhibited TE reductions on Rap treatment (Table 1, row 4, cols. 3–5), and the *ded1-cs* and *ded1-ts* mutations conferred little or no TE reductions for the 147 mRNAs translationally down-regulated by Rap (Figure S2D, cols. 2–3). Moreover, Rap treatment did not evoke the formation of PBs or SGs [35,36], suggesting the absence of Ded1 condensation. It cannot be excluded, however, that a small reduction in Ded1 function on the inhibition of TORC1 synergistically acted with the impairment of another factor to reduce the translation of some of the 46 Ded1-hyperdependent mRNAs that were affected by Rap treatment (Table 1, row 5, col. 1). eIF4G is an obvious candidate for such a factor in view of evidence that eIF4G stability is reduced by Rap treatment [23] in a manner involving its interaction with Ded1 [22]; however, we found that the group of mRNAs translationally impaired by Rap showed no change in relative TE upon the depletion of eIF4G1 in a strain lacking eIF4G2 in the profiling data from Zinshteyn et al. [31] (Figure S4B). Further work is required to uncover the molecular basis for the TE reductions conferred by Rap.

In conclusion, the evidence we uncovered here for the reduced translation of subsets of Ded1-hyperdependent mRNAs in the stress conditions of glucose starvation and heat-shock supports proposals that the dissociation of Ded1 from mRNA 5′UTRs [20] and the condensation of Ded1 and of mRNAs bound to Ded1 under these stress conditions [19] contribute to the translational repression of mRNAs with heightened Ded1-dependence in stressed cells. It is also possible that covalent modifications of Ded1 occur in stressed cells that lead to reductions in its unwinding activity. However, our results also indicate that only particular subsets of Ded1-hyperdependent mRNAs are strongly repressed under these conditions and support the possibility that such repression may additionally depend on inhibiting the functions of other initiation factors on these mRNAs, notably eIF4B or eIF4A [20], by a combinatorial mechanism (Figure 6). Finally, large proportions of mRNAs translationally down-regulated by glucose starvation or heat-shock are not Ded1-hyperdependent mRNAs, implicating the negative regulation of other factors besides Ded1 in their translational repression.

## Figures and Tables

**Table 1:** Summary of overlaps between mRNA groups translationally impaired by stresses versus *ded1* mutations.

1. Mutation or Condition	2. % of mRNAs in TE_Down_*ded1-cs* or TE_Down_*ded1-ts* Group ^1^	3. % Ded1-Hyperdep. mRNAs (*ded1-cs*) Impaired by Condition ^2^	4. % Ded1-Hyperdep. mRNAs (*ded1-ts*) Impaired by Condition ^2^	5. % 258 Most Ded1-Hyperdep. mRNAs Impaired by Condition ^2^
1. *DED1-IDRm*	74% (of 282)	14% (of 1505)	21% (of 460)	25% (of 258)
2. −Glu	42% (of 520)	14% (of 1505)	18% (of 460)	21% (of 258)
3. HS (42°/40°)	28% (of 258)	4.4% (of 1505)	8.7% (of 460)	9.3% (of 258)
4. Rap	31% (of 147)	3% (of 1505)	2.4% (of 460)	2.7% (of 258)
5. HS (42°/30°)	47% (of 431)	12.9% (of 1505)	28% (of 460)	36% (of 258)

^1^ Proportion of TE_down mRNAs for each mutation/condition in column 1 (total number in parenthesis) found in the group of 1566 mRNAs belonging to either the TE_down_*ded1-cs* or TE_down_*ded1-ts* groups showing TE reductions of >1.5-fold at FDR < 0.05 in each *ded1* mutant versus WT. ^2^ Proportion of the Ded1-hyperdependent mRNAs (total number in parenthesis) translationally impaired by the condition/mutation in column 1.

## Data Availability

The study analysed public datasets available in the GEO repositories.

## Supplementary Materials

The following are available online at https://www.mdpi.com/article/10.3390/microorganisms9122413/s1, Figure S1: Ded1-hyperdendent mRNAs generally do not exhibit TE changes as an immediate response to glucose starvation, Figure S2: Rapamycin treatment elicits translational changes largely distinct from those conferred by *ded1-ts* and *ded1-cs* mutations, Figure S3: A significant fraction of Ded1-hyperdependent mRNAs identified in *ded1-ts* cells is translationally down-regulated by heat-shock of WT cells at 42 °C versus 40 °C, Figure S4: Depletion of eIF4G does not preferentially reduce the TEs of subsets of Ded1-hyperdependent mRNAs translationally down-regulated by glucose starvation, the DED1-IDRm mutation, or heat-shock at 42 °C versus 30 °C, nor the mRNAs translationally downregulated by Rap, Figure S5: The mRNAs translationally impaired by the *DED1-IDRm* mutation are enriched for those impaired by heat-shock at 42 °C versus 30 °C but not by heat-shock at 42 °C versus 40 °C, Figure S6. Ded1-hyperdependent mRNAs exhibit greater than average 5′UTR Ded1 occupancies, Table S1: Ribosome profiling datasets used in current study. File S1. Translation of Ded1-hyperdependent mRNAs is highly similar among the non-stressed WT control cells for the different ribosome profiling datasets.
