# Supplementary material for: Down-Regulation of Yeast Helicase Ded1 by Glucose Starvation or Heat-Shock Differentially Impairs Translation of Ded1-Dependent mRNAs
Source: Microorganisms. 2021 Nov 23;9(12):2413. doi: 10.3390/microorganisms9122413 (PMC8706886; doi:10.3390/microorganisms9122413)
Supplement: Supplementary file 1 [file microorganisms-09-02413-s001.zip › microorganisms-1428881-supplementary.pdf]

SUPPLEMENTARY MATERIAL FOR:

Down-Regulation of Yeast Helicase Ded1 by Glucose Starvation or Heat-Shock Differentially Impairs Translation of Ded1-Dependent mRNAs

SUPPLEMENTARY FIGURE LEGENDS

Fig. S1

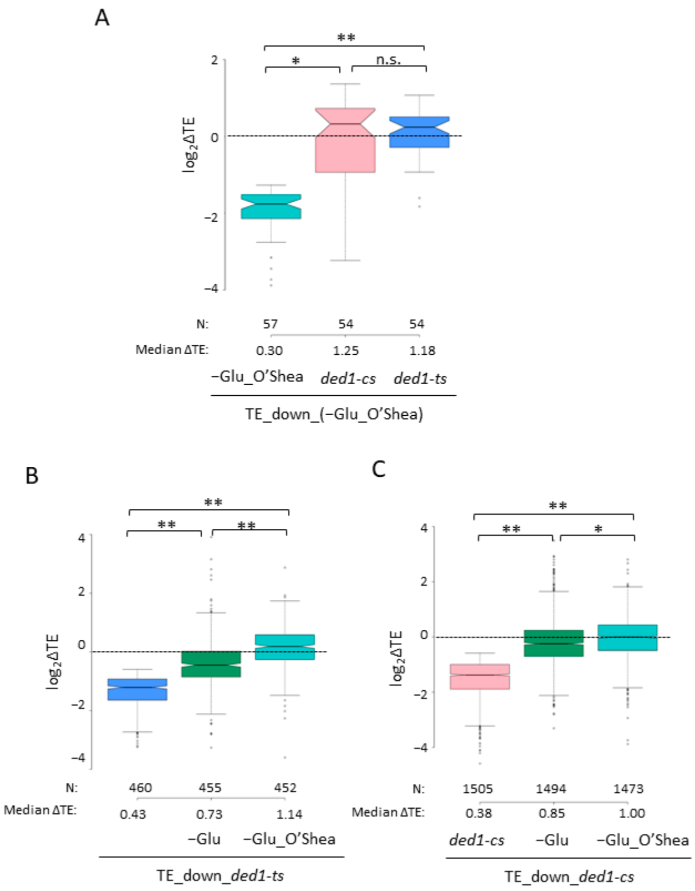

Figure S1. Ded1-hyperdendent mRNAs generally do not exhibit TE changes as an immediate response to glucose starvation. (A-C) Notched box plots showing log$_2$ fold-changes in TE (log$_2$ΔTE) after 15 min of glucose starvation, or in response to the *ded1-cs* or *ded1-ts* mutations, for (A) 57 mRNAs showing significant TE changes after 15 min of glucose starvation (TE_down_(-Glu_O'Shea)), (**B**) the 460 mRNAs showing ≥1.5-fold reductions in TE in *ded1-ts* cells at FDR < 0.05, TE_down_*ded1-ts* mRNAs, and (**C**) the 1505 mRNAs showing ≥1.5-fold reductions in TE in *ded1-ts* cells at FDR < 0.05, TE_down_*ded1-cs* mRNAs. *P*-values computed from Mann–Whitney *U*-test are given (*, $P < 10^{-12}$; **, $P < 10^{-15}$; n.s., not significant).

Fig. S2

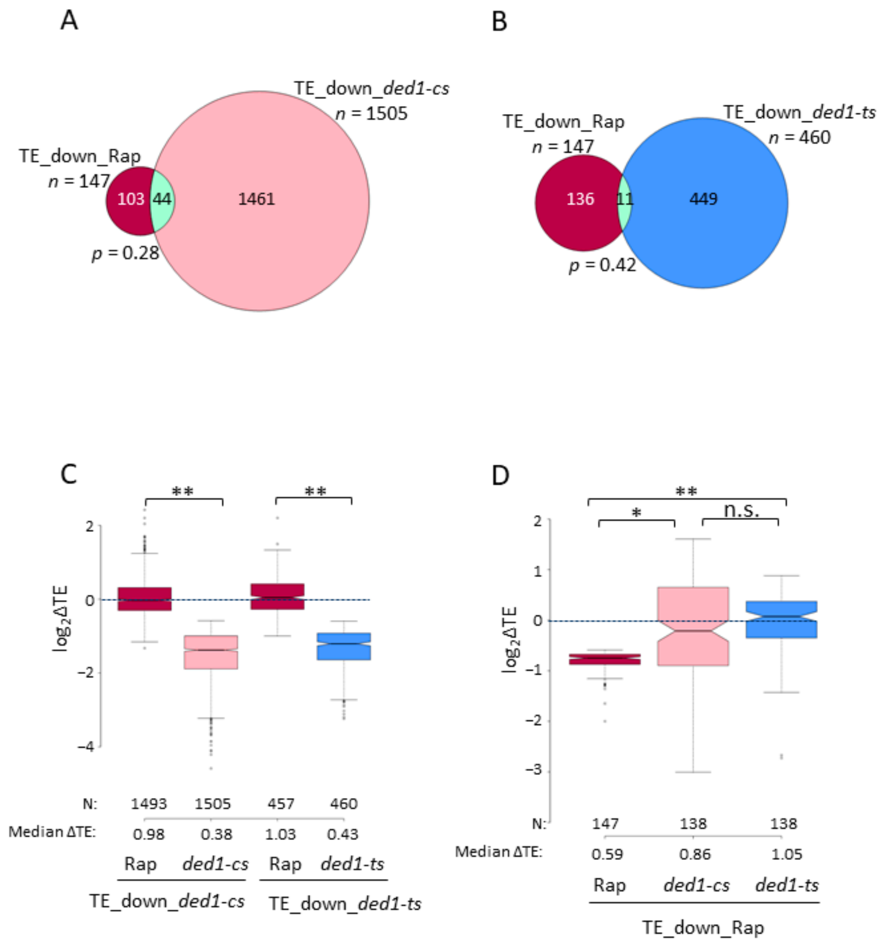

Figure S2. Rapamycin treatment elicits translational changes largely distinct from those conferred by *ded1-ts* and *ded1-cs* mutations. (A-B) Overlap between 147 mRNAs exhibiting ≥1.5-fold reductions in TE at FDR < 0.05 on rapamycin treatment (TE_down_Rap) and either the TE_down_*ded1-cs* group (**A**) or the TE_down_*ded1-ts* group of mRNAs (**B**). **(C-D)** Notched box plots showing $\log_2$ fold-changes in TE in response to Rap, *ded1-cs,* or *ded1-ts* for the 1505 TE_down_*ded1-cs* mRNAs (cols. 1-2) or the 460 TE_down_*ded1-ts* mRNAs (cols. 3-4). **(D)** $\log_2$ fold-changes in TE in response to Rap, *ded1-cs,* or *ded1-ts* for the 147 TE_down_Rap mRNAs. *P*-values computed from Mann–Whitney *U*-test are given (*, $P < 10^{-9}$; **, $P < 10^{-15}$; n.s., not significant).

Fig. S3

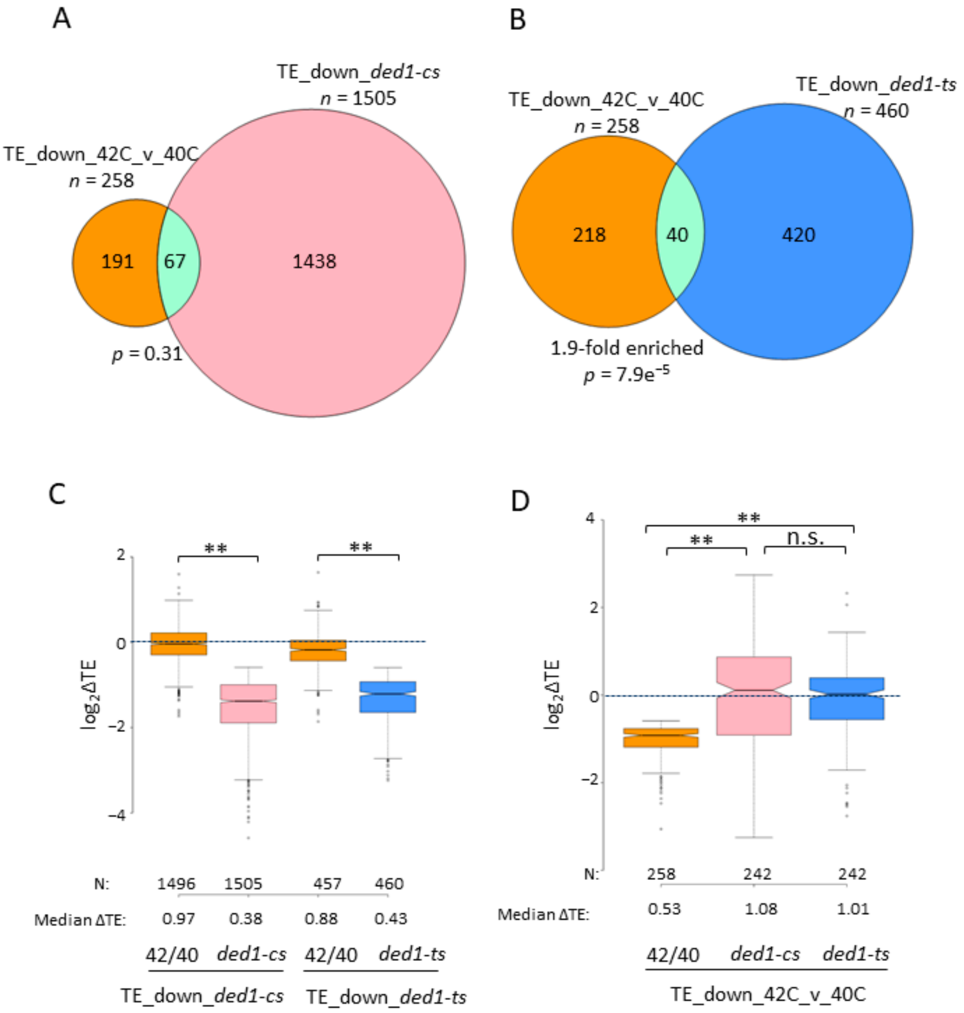

Figure S3. **A significant fraction of Ded1-hyperdependent mRNAs identified in *ded1-ts* cells is translationally down-regulated by heat-shock of WT cells at 42 °C versus 40 °C.** (A-B) Overlap between TE_down_42C_v_40C mRNAs and either TE_down_*ded1-cs* (**A**) or TE_down_*ded1-ts* (**B**) mRNAs. One outlier was omitted from the plot to expand the y-axis scale. **(C)** Log_2 fold changes in TE in response to heat-shock of WT cells at 42°C versus 40 °C (42C/40C), *ded1-cs*, or *ded1-ts* for the 1505 TE_down_*ded1-cs* mRNAs (cols. 1-2) or 460 TE_down_*ded1-ts* mRNAs (cols. 3-4). **(D)** Log_2 fold-changes in TE in response to heat-shock at 42 °C vs. 40 °C, *ded1-cs*, or *ded1-ts* for the 258 TE_down_42C_v_40C mRNAs. *P*-values computed from Mann–Whitney *U*-test are given (**, $P < 10^{-15}$; n.s., not significant).

Fig. S4

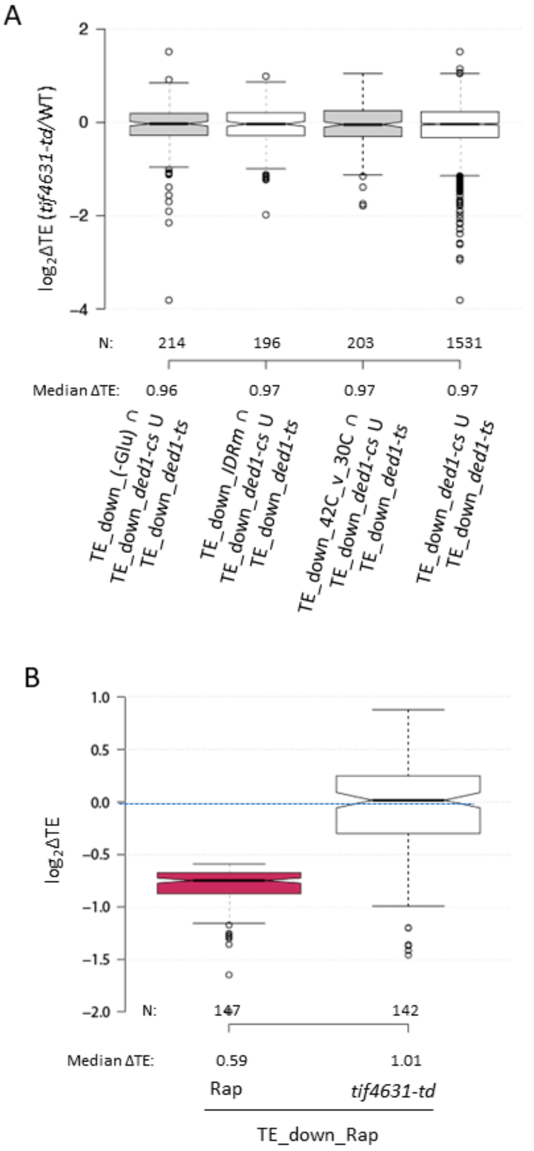

Figure S4. **Depletion of eIF4G does not preferentially reduce the TEs of subsets of Ded1-hyperdependent mRNAs translationally down-regulated by glucose starvation, the *DED1-IDRm* mutation, or heat-shock at 42°C versus 30°C, nor the mRNAs translationally downregulated by Rap.** (**A**) Log_2 fold- changes in TE conferred by depletion of eIF4G in a *tif4631-td tif4632Δ* mutant for the 1566 mRNAs showing 1.5-fold TE reductions in response to either *ded1-cs* or *ded1-ts* mutations (col. 4) or the subsets of these mRNAs that also show TE reductions in response to either glucose starvation (217 mRNAs, col. 1), the *DED1-IDRm* mutation (201 mRNAs, col. 2), or heat shock at 42°C versus 30°C (203 mRNA, col. 3). **(B)** Log_2 fold changes in TE conferred by Rap (col. 1) or depletion of eIF4G in a *tif4631-td tif4632Δ* mutant (col. 2) for the 147 TE_down_Rap mRNAs.

Fig. S5

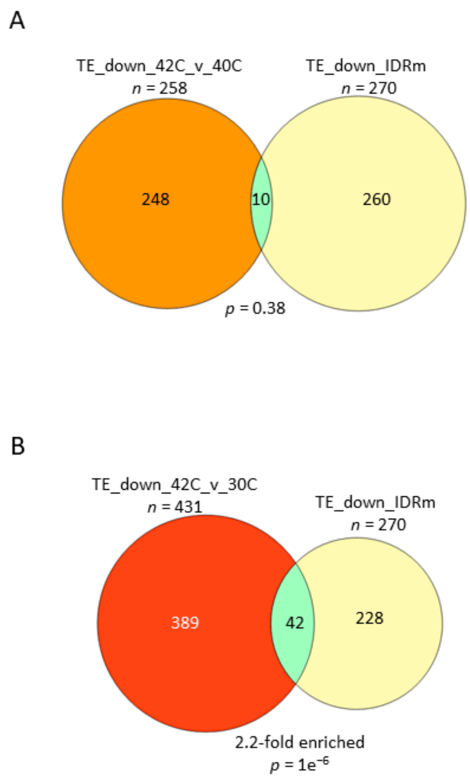

Figure S5. The mRNAs translationally impaired by the *DED1-IDRm* mutation are enriched for those impaired by heat-shock at 42 °C versus 30 °C but not by heat-shock at 42 °C versus 40 °C. (**A–B**) Overlap of TE_down_*IDRm* mRNAs with either TE_down_42C_v_40C (A) or TE_down_42C_v_30C (B) mRNAs.

Fig. S6

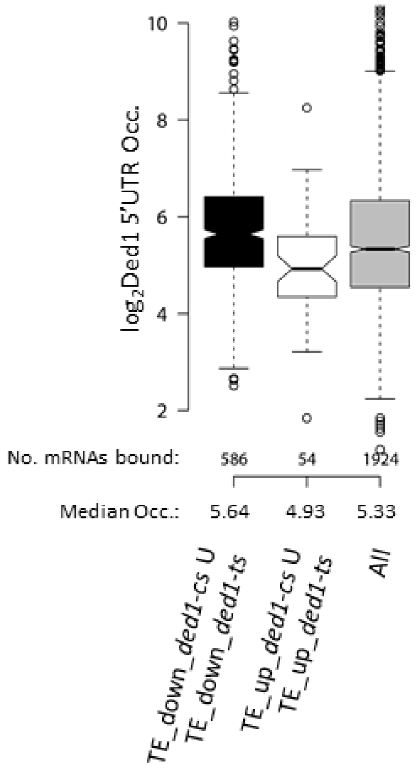

Figure S6. **Ded1-hyperdependent mRNAs exhibit greater than average 5′UTR Ded1 occupancies.** UV-crosslinking RNA immunoprecipitation (CRAC) data for Ded1 obtained from control, non-stressed cells (taken from Bresson et al. (2020)) was analyzed for the 1566 Ded1-hyperdependent mRNAs (TE_down_*ded1-cs* U TE_down_*ded1-ts*, described in Fig. 5, cols. 9-12), the 1756 Ded1-hypodependent mRNAs (showing TE increases of >1.5-fold at FDR<0.05 in either *ded1-cs* or *ded1-ts* cells), and all 5423 mRNAs analyzed in this study. The log2 reads per million in the 5′UTR or beginning of the ORF bound to Ded1 are plotted. Note that only 3% of the Ded1-hypodependent mRNAs showed detectable Ded1 binding, compared to 37% and 35% of the Ded1-hyperdependent and all mRNAs, respectively, implying a much lower median Ded1 occupancy than indicated here for the hypodependent group.

Table S1. Ribosome profiling datasets used in current study.

Strain background	Growth conditions	Source	GEO accession numbers	CHX treatment	values from DESeq2 ($log_2ΔTE$)
Σ1278b	3h Glu starvation in YP medium	(Vaidyanathan et al. 2014); GSE51532	GSM1247439<br>GSM1247440<br>GSM1247441<br>GSM1247442<br>GSM1247443<br>GSM1247444<br>GSM1247445<br>GSM1247446<br>GSM1247447<br>GSM1247448<br>GSM1247449<br>GSM1247450	Yes	−Glu
BY4741	Treatment with 12.5 nM Rap in YPD medium for 30 min	(Nedialkova and Leidel 2015); GSE67387	GSM1646015<br>GSM1646016<br>GSM1646017<br>GSM1646051<br>GSM1646052<br>GSM1646053<br>GSM1646027<br>GSM1646028<br>GSM1646029<br>GSM1646057<br>GSM1646058<br>GSM1646059	Yes	Rap
W303	Heat-shock of WT for 10 min at 42°C vs 40°C in YPD medium	(Iserman et al. 2020); GSE131176 (ribo) & GSE141029 (mRNA)	GSM3765213<br>GSM3765214<br>GSM3765215<br>GSM3765216<br>GSM4192627<br>GSM4192628<br>GSM4192629<br>GSM4192630	No	42C/40C
W303	Heat-shock of WT for 10 min at 42°C vs 30°C in YPD medium	(Iserman et al. 2020); GSE131176 (ribo) & GSE141029 (mRNA)	GSM3765211<br>GSM3765212<br>GSM3765215<br>GSM3765216<br>GSM4192625<br>GSM4192626<br>GSM4192629<br>GSM4192630	No	42C/30C
W303	*DED1-IDRm* vs. WT at 42°C in YPD medium	(Iserman et al. 2020);	GSM3765215<br>GSM3765216	No	*IDRm*/WT

		GSE131176 (ribo) & GSE141029 (mRNA)	GSM3765221<br>GSM3765222<br>GSM4192629<br>GSM4192630<br>GSM4192635<br>GSM4192636		
BY4741	WT shifted to SC medium lacking glucose for 15 min	(Zid and O'Shea 2014): GSE56622	GSM1365918<br>GSM1387087<br>GSM1365925<br>GSM1387091<br>GSM1365921<br>GSM1387089<br>GSM1365928<br>GSM1387093	Yes	−Glu_O'Shea
S288C	*tif4631-td (tif4632Δ::kanMX6 P$_{CUP1}$-UBI-R-DHFRts-HA-tif4631-td* vs. WT (*tif4632Δ::kanMX6 TIF4631),* in SC+2% raffinose, 2% galactose at 36°C for 2h	Zinshteyn et al. (2017): GSE87614	GSM2335482<br>GSM2335484<br>GSM2335486<br>GSM2335488<br>GSM2335490<br>GSM2335492<br>GSM2335494<br>GSM2335496	Yes	*tif4631-td*
BY4741	*ded1-cs* and WT shifted to SC medium at 15°C for 10 min	(Sen et al. 2019); GSE 111255	GSM3027242<br>GSM3027243<br>GSM3027244<br>GSM3027245<br>GSM3027248<br>GSM3027249<br>GSM3027250<br>GSM3027251	Yes	*ded1-cs*
BY4741	*ded1-ts* and WT shifted to SC medium at 37°C for 2h	(Sen et al. 2019); GSE 111255	GSM3027254<br>GSM3027255<br>GSM3027256<br>GSM3027257<br>GSM3027260<br>GSM3027261<br>GSM3027262<br>GSM3027263	Yes	*ded1-ts*
BY4741	*tif1-ts* and WT shifted to SC medium at 37°C for 1h	(Sen et al. 2015); GSE 66411	GSM1622004<br>GSM1622005<br>GSM1622006<br>GSM1622007<br>GSM1622008<br>GSM1622009<br>GSM1622010<br>GSM1622011	Yes	*tif1-ts*

			GSM1622012<br>GSM1622013<br>GSM1622014<br>GSM1622015<br>GSM1622016<br>GSM1622017<br>GSM1622018<br>GSM1622019		
BY4741	*tif3Δ* and WT shifted to SC medium at 37°C for 1h	(Sen et al. 2016); GSE 81966	GSM2178815<br>GSM2178816<br>GSM2178817<br>GSM2178818<br>GSM2178819<br>GSM2178820<br>GSM2178821<br>GSM2178822	Yes	*tif3Δ*
